# The impact of adverse childhood experiences on EMG reactivity: A proof of concept study

**DOI:** 10.1371/journal.pone.0216657

**Published:** 2019-05-09

**Authors:** Anne Marsman, Rosan Luijcks, Catherine Vossen, Jim van Os, Richel Lousberg

**Affiliations:** 1 Department of Psychiatry and Psychology, Maastricht University, Maastricht, The Netherlands; 2 Department of Anesthesiology and Pain Medicine, Maastricht University Medical Centre, Maastricht, The Netherlands; 3 Department of Psychiatry, UMC Utrecht Brain Centre, Utrecht, The Netherlands; 4 King’s College London, King’s Health Partners, Department of Psychosis Studies, Institute of Psychiatry, London, United Kingdom; Temple University, UNITED STATES

## Abstract

Adverse childhood experiences (ACE), such as emotional or physical abuse, can produce a lasting effect on the individual. The aim of this study was to investigate how ACE may impact electromyography (EMG) activity of the trapezius muscle in a novel experimental stress paradigm, in a sample of 120 healthy participants. The stress paradigm consisted of a memory task, in which participants were asked to memorize and recall as many words as possible, displayed on a screen. The study protocol included 2 identical experimental sessions (T_0_ = 0 and T_1_ = 6 months). EMG activity was analyzed using multilevel regression analysis. EMG activity was higher during the memory task compared to baseline, supporting the validity of the experimental EMG-stress paradigm. In addition, the EMG increase was attenuated during the second session. Analyses were indicative for a moderating effect of ACE on stress-induced EMG activity: higher ACE scores resulted in greater EMG reactivity. These associations were apparent for early ACE exposure (0–11 years) as well as for later exposure (12–17 years). The association between ACE and EMG reactivity remained significant but was much weaker at T_1_ in comparison to T_0_, likely because of reduced unpredictability and uncertainty related to the experiment.

In conclusion, this study showed that enduring liabilities occasioned by ACE in a non-clinical population can be studied using an experimental paradigm of EMG stress reactivity, contingent on the level of predictability of the stressor.

## Introduction

There is extensive evidence that adverse childhood experiences (ACEs), such as emotional or physical abuse, can have long lasting effects on the individual [1–3]. Several studies have revealed that ACEs negatively impact neurobiological processes, immunological parameters and autonomic, endocrine and metabolic systems [4–7]. There is also evidence that exposure to early life adversity increases stress sensitivity later in life, which in turn is thought to increase vulnerability to develop mental disorders such as depression and psychosis following adult stressful life events [2, 8]. Additionally, in the Adverse Childhood Experiences Study (ACE Study)–a large epidemiological research project conducted in the United States to assessing the long-term effects of childhood abuse on adult health problems–a link between many types of childhood adversity and adult onset of somatic and mental health disorders was apparent [9, 10]. In addition, there is evidence of a dose-response relationship in the association between ACEs and later mental health problems [9, 11, 12].

The experience of early life adversity can be highly variable, for instance with regard to factors such as type of maltreatment and duration, predictability and severity of the stressor. Regardless of these factors however, ACEs by nature share the commonality of being stressful and stress inducing [12].

Although it may be hypothesized that ACEs considered as ‘more severe’ (e.g. sexual abuse as opposed to financial problems) result in more disadvantage, findings of the ACE Study showed that ten different types of adversity exerted equivalent negative effects [9]. One explanation for this finding was offered by Dong and colleagues, who studied data from the larger ACE Study and demonstrated that ACEs often co-occur and should thus be viewed as a complex set of highly interrelated experiences, rather than stand-alone events [13]. Thus, when assessing the impact of ACE, the authors suggest that the experiences should not be assumed to be isolated events during childhood but rather as co-occuring with other types of adversity [13].

Another explanation for the apparent equivalence of negative effects pertaining to different ACEs is that the developing brain reacts similarly to different types and degrees of adversity because they share the common feature of unpredictability [12]. During childhood, the unpredictability of an event (e.g. not knowing when, why or from where an emotional or physical event may arise) may be most impactful. Evidence suggests that unpredictability associated with ACE exposure may be particularly important for younger children [14]. This is compatible with literature suggesting that ACE exposure in early childhood (0–11 years) is associated with more harmful effects than exposure at an older age (12–17 years) [1, 3].

Animal research on chronic unpredictable stress supports the notion that it is particularly the degree of unpredictability of stress that mediates its harmful consequences. In one study, rats were exposed to unpredictable mild stressors for three weeks [15]. During this time, at unpredictable moments, one of three stressors was applied: cage rotation, one-day food restriction or thirty minutes exposure to strobe lights. After three weeks, effects on the brain were evaluated. The group that was exposed to unpredictable mild stressors showed significant changes in hippocampal receptors–a brain area associated with emotion and stress regulation. The control group that experienced no stress showed no brain changes [15]. The authors followed up this finding and found that when stress is completely predictable, even if more severe, brain changes were much less apparent compared to three weeks of moderate unpredictable stress [12].

While many studies have demonstrated the long-lasting negative effects of adverse childhood experiences on future health and well-being, relatively little is known about etiological underlying (biopsychosocial) mechanisms. However, there is widespread consensus on two key aspects related to the impact of stress. First, the concept of ‘stress’ itself is best conceptualized as a theoretical construct that is not directly measurable [16]. Second, stress per definition induces (psycho)physiological changes, such as in heart rate, cortisol levels and muscle tension levels, varying from hardly noticeable to extreme [17, 18]. In this context, Luijcks and coworkers showed that trapezius muscle stress reactivity is a good candidate for examining the impact of ACE on a physiological stress-related outcome [19–21]. These authors examined the association between ACE and muscle activity in a recently developed experimental stress paradigm, in which the focus was on anticipatory cognitive stress preceding a single unpredictable and uncontrollable electrical painful stimulus. They found that anticipatory electromyography (EMG) stress reactivity during the experiment, indexed as trapezius muscle activity, was consistently stronger in participants with higher levels of ACE. It was furthermore shown that early childhood ACE (0–11 years) had a stronger moderating effect than adolescent ACE (12–17 years) [21]. This finding was true for both session 1 at the beginning of the study and session 2 at 6 months, confirming the reliability of the experiment.

In the present study, we wish to extend the findings by Luijcks and colleagues on the influence of ACE on EMG activity to a different experimental stress paradigm. This paradigm consisted of an EMG baseline measurement followed by a novel memory task in which participants were instructed to memorize and recall as many words as possible, displayed on a screen. In contrast to the experimental stress experiment used by Luijcks and coworkers, which included a physical stressor (an electrical shock), in the current study, the stressor impacting trapezius muscle activity was cognitive by nature. Previous research has shown that cognitive tasks such as the Stroop color word test and mental arithmetic tasks successfully induce mental stress, resulting in changes in EMG reactivity of the trapezius muscle [20, 22]. In the current paradigm we propose a newly designed memory task for stress induction that is easier to apply and generalizes better to daily life stress than for instance the Stroop test does.

In order to investigate the role of predictability in the experiment, the study protocol included 2 identical experimental sessions for each participant (T_0_ = 0 months, session 1, and T_1_ = 6 months, session 2). Since the experimental stress memory task had not been used before, validation of the experimental EMG-stress paradigm was the first objective of the study. To this end, two effects were tested, one main condition effect (baseline vs memory task) and one condition*session interaction effect. The a priori hypothesis was the existence of a significant positive condition effect (higher EMG reactivity during the memory task compared to baseline) as well as a negative condition*session effect (lower reactivity during session 2 compared to session 1). Furthermore, a significant main effect of age was expected, increasing age resulting in lower EMG activity [23]. The following set of variables were added as potential confounders: NEO-neuroticism scale, STAI (state version), perceived stress (PSS), educational level and life events in the previous year. In the method section, these variables are explained in more detail.

The second objective was to further explore the association between ACE and stress-related trapezius muscle activity. To this end, we investigated the cross-experiment generalizability of the finding by Luijcks and colleagues that ACE influences EMG stress reactivity in a cognitive stress experiment. Based on previous research, it was hypothesized that exposure to ACE, particularly those occurring during early childhood, would be associated with increased trapezius muscle activity during the memory task. Furthermore, it was hypothesized that the moderating effect of ACE would be smaller 6 months later, given a reduction in unpredictability during the second session (compared to the first session).

Since multilevel regression analysis was used to test the hypotheses [21], we were able to also include random effects. For all models, we hypothesized a random intercept (representing general EMG variability between participants), a random condition effect (indicating differential task reactivity) as well as a random session effect (demonstrating between-session variability).

## Materials and method

### Ethics statement

The experiment was part of a larger study that evaluated psychophysiological reactivity as a predictor of change in pain and depressive complaints. The original study was conducted according to the principles of the Declaration of Helsinki and was approved by the medical ethics committee of the Academic Hospital Maastricht and Maastricht University (METC azM/UM, NL40284.068.12/METC 12-3-015). All participants provided written informed consent before the start of the experiment.

### Participants

The sampling frame was a general population sample derived from the population living in the city of Maastricht, the Netherlands, who had responded to flyers that were handed out in several public places. One hundred and twenty participants (78 women and 42 men) participated in the study. Of these, 114 were right-handed. Age ranged from 18 to 65 years (mean 40.5, SD 17.1). Exclusion criteria were structural use of antipsychotics, antiepileptics or anxiolytics during the past year or structural use of alcohol in excess of 7 u/day. Participants were asked to refrain from alcoholic beverages the evening before the experiment, and to refrain from caffeine-containing consumptions three hours prior to the experiment. Participants received €50 for participating in the study.

### Procedure

Upon arrival in the laboratory, participants were first asked to complete a set of questionnaires, after which EMG and ECG electrodes were attached. EMG electrodes were attached on the left and right trapezius muscles (LTM and RTM).

The baseline measurement was 5 minutes, in which participants were instructed to sit as still as possible, with their eyes open. After the baseline, participants continued with the memory task. Participants were instructed to look at a screen on which words appeared, and were asked to memorize as many words as possible. In a fixed (category) semi-randomized order, 40 words were presented on the screen, each word displayed for 2 seconds. The 40 words were divided into 4 categories: positive connotation, negative connotation, pain-related and neutral. Each category contained 10 words. After the presentation of 40 words, participants had 1 minute to recall all memorized words. This procedure was repeated 3 times, and each time participants were asked to perform as best as they could. The study protocol included an identical follow-up measurement 6 months after the first session.

### Psychophysiological recordings

EMG activity was recorded from the left and right upper trapezius muscle. Recordings were conducted in an electrically and sound-shielded cubicle (7.1 m^2^), using Ag/AgCl electrodes centered on a point 2 cm lateral to the midpoint between the acromion process and spinous process of the seventh cervical vertebra. A reference electrode was placed over the spinous process of the seventh cervical vertebra. ECG activity was recorded with a standard 3-lead ECG. Conductive paste (Ten20) was used to fix all electrodes. Brainvision BrainAmp Research Amplifier was used for all recordings. ECG and EMG were sampled with 1000 Hz.

### Psychological measurements

ACEs were assessed with a questionnaire developed within the context of the FP7 EU-GEI project (European Network of National Schizophrenia Networks Studying Gene-Environment Interactions) [24]. The Childhood Experiences of Care and Abuse questionnaire comprises 15 yes/no questions. Examples of items are: ‘Were your basic needs ever neglected?’ and: ‘Was there an adult person you could talk to about problems or your feelings?’ In addition to these items, issues such as the presence of financial problems in the family, the occurrence of sexual abuse, and so on, were assessed. The questionnaire covers two exposure periods: the first period includes exposure between 0 and 11 years (early childhood), the second period between 12 and 17 years (adolescence). Prior research has shown adequate internal consistency coefficients for both age periods [21]. The maximum score in each age period was 15, the maximum score for the entire questionnaire was 30. The sum of scored events for both age periods together ranged from 0 to 14 (mean 3.4, SD 3.4). For the exposure period of 0 to 11 years, the sum of scored events ranged from 0 to 9 (mean 1.8, SD 1.9), whereas for the exposure period of 12 to 17 years, the sum of scored events ranged from 0 to 8 (mean 1.6, SD 1.8).

**Neuroticism** was assessed with the Revised Neuroticism-Extraversion-Openness Personality Inventory (NEO-PI-R). The NEO-PI is a 240-item self-rating questionnaire that measures five major personality dimensions. Items are scored on a five-point Likert scale, ranging from 1 (disagree strongly) to 5 (agree strongly). The questionnaire has been validated extensively [25] and is widely used to operationalize the five-factor model of personality [26]. From the NEO-PI-R a total neuroticism score was calculated, representing a tendency or predisposition to experience negative affective states such as anxiety, depression, anger and impulsiveness. The total score ranged from 80 to 200 (mean 130, SD 22.5).

**State anxiety** was assessed with the State-Trait Anxiety Inventory (STAI), a commonly used measure of trait and state anxiety [27]. The questionnaire consists of 20 items assessing trait anxiety and 20 for state anxiety. The State Anxiety scale assesses the current anxiety state using items such as ‘I am tense; I am worried’ and ‘I feel calm; I feel secure’. Items are rated on a 4-point Likert scale ranging from 1 (almost never) to 4 (almost always). Higher scores indicate greater anxiety. The total score ranged from 20 to 77 (mean 35.7, SD 10.3).

**Perceived Stress** was assessed by the Perceived Stress Scale (PSS), the most widely used instrument with acceptable psychometric properties for measuring the perception of stress in daily life [28]. The PSS is a 10-item questionnaire, asking about feelings and thoughts during the last month. Items such as ‘In the last month, how often have you been upset because of something that happened unexpectedly?’ are rated on a 5-point Likert scale ranging from 0 (never) to 4 (very often). Total score ranged from 20 to 39 (mean 11.3, SD 6.2).

**Recent life stressors** were assessed by asking participants whether or not they had experienced one or more events that impacted their daily lives in the previous year. A few examples of life events from the Holmes-Rahe Stress Inventory [29] were provided to give participants an idea of what should be regarded a ‘life event’. In order to keep the experiment as short as possible the assessment only consisted of a yes/no question, instead of using the entire Holmes-Rahe Stress Inventory. Overall, 86% of the participants experienced at least one life event in the previous year.

### Offline data processing

EMG data was filtered offline (low pass 0.5 Hz, high pass 250 Hz, 50 Hz notch filter) and segmented into segments of 2000 milliseconds. Raw data were visually inspected for artifacts using software (BrainVision Analyzer 2.0) and excluded from further analyses when detected. EMG activity was corrected for ECG activity. Using regression analysis, the variance due to ECG activity was removed from the uncorrected EMG variable. Next, for each 2000 ms segment, the root mean square (RMS) value was calculated followed by a 10log logarithmic transformation to preserve a normal distribution. Due to hardware memory limitations, the number of total segments had to be restricted in order to perform the multilevel regression analysis. Consequently, for each participant, the 5 minutes baseline period was divided into 30 segments, each with a duration of 10 seconds.

The memory task was also divided into 10-second segments (resulting in 24 segments), each segment containing 5 consecutive words.

### Statistical analysis

The EMG dataset had a hierarchical structure consisting of repeated time segments (level 1), nested within individuals (levels 2). Given this structure, multilevel regression analysis was performed, in which EMG activity of the LTM and RTM served as the dependent variable in all models.

As outlined above, we first executed a series of validation analyses. We tested a main condition effect contrasting baseline (coded “0”) with the memory task (coded “1”) and a condition*session interaction effect (where session was coded “0” at T_0_ and “1”at T_1_). Additionally, age (in years) and sex were included as predictor variables, as well as the NEO-neuroticism scale, STAI (state version), perceived stress (PSS), educational level and life events in the previous year.

In the next series of models, ACE score for the early childhood period and the adolescent period were included as key predictor variables, in separate models. The third-order interaction between ACE, session and condition was of main interest in these models. As argued in the introduction, we expected negative coefficients of this interaction term (representing a decreased moderating effect of ACE during the second session), particularly in the model including early childhood ACE.

Initially, we also planned to analyze the impact of the 4 different word categories (see above) on EMG reactivity. However, the multilevel analyses could not accommodate the extra number of records required for these analyses.

In order to test which covariance structure should be applied for this dataset, various covariance structures were tested. In agreement with earlier work with comparable experiments [21, 30], AR1 yielded the best fit. All models were run with a random intercept, random condition and random session effect. All statistical analyses were performed using SPSS 24.0. P-values ≤ 0.05 were considered to be statistically significant.

## Results

A total of 120 participants were enrolled (78 female, 42 male). At the second experimental session, there was attrition of 15 participants. Thus, the multilevel dataset consisted of N = 120 participants for session 1 and N = 105 participants for session 2. General characteristics of the sample are displayed in Table 1. Independent sample t-tests and Chi-square tests revealed that there was no significant difference between variables at the first and second measurement (all p > .653). Approximately 40% of all participants experienced 2 or more adverse events.

**Table 1:** General characteristics and descriptive statistics of the study population.

Variables	T_0_ (N = 120)	T_1_ (N = 105)
Age, mean (SD) min-max	39.70 (17.06) 18–66	41.01 (16.99) 18–66
Gender (%)		
Male	35.3	36.1
Female	64.7	63.9
Educational level (%)		
Lower	32.3	32.3
Middle	26.1	26.8
Higher	41.6	40.9
Life event(s) past year (%)		
No	13.5	14.4
Yes	86.5	85.6
Early ACE		
None	31.1	30.6
1	24.4	24.7
2	17.2	17.0
> 2	27.3	27.7
Adolescent ACE		
None	34.5	34.6
1	26.9	26.3
2	13.4	15.4
> 2	25.2	23.7
STAI-state, mean (SD) min-max	35.89 (10.17) 20–77	35.45 (10.39) 20–77
PSS-score, mean (SD) min-max	1.13 (.61) .20–3.90	1.12 (.62) .20–3.90
NEO-neuroticism, mean (SD) min-max	129.86 (22.04) 80–200	130.10 (23.06) 80–200

ACE, Adverse Childhood Experience; STAI, State-Trait Anxiety Inventory, state-subscale; PSS, Perceived Stress Scale; NEO, Neuroticism-Extraversion-Openness Personality Inventory, neuroticism-subscale.

### Validation analyses: EMG activity during baseline and memory task

As hypothesized, a significant EMG increase was demonstrated during the memory task compared to baseline, for both left (t = 3.861, p < .001) and right (t = 4.788, p < .001) trapezius muscle. Contrary to our expectations, we did not observe a significant main association with age (p = .099). We did find a positive association between EMG and education level for both left (t = 2.608, p = .011) and right (t = 2.062, p = .042) trapezius muscle activity, indicating that people with higher educational levels show increased EMG activity compared to people with lower educational levels.

**Fig 1:**
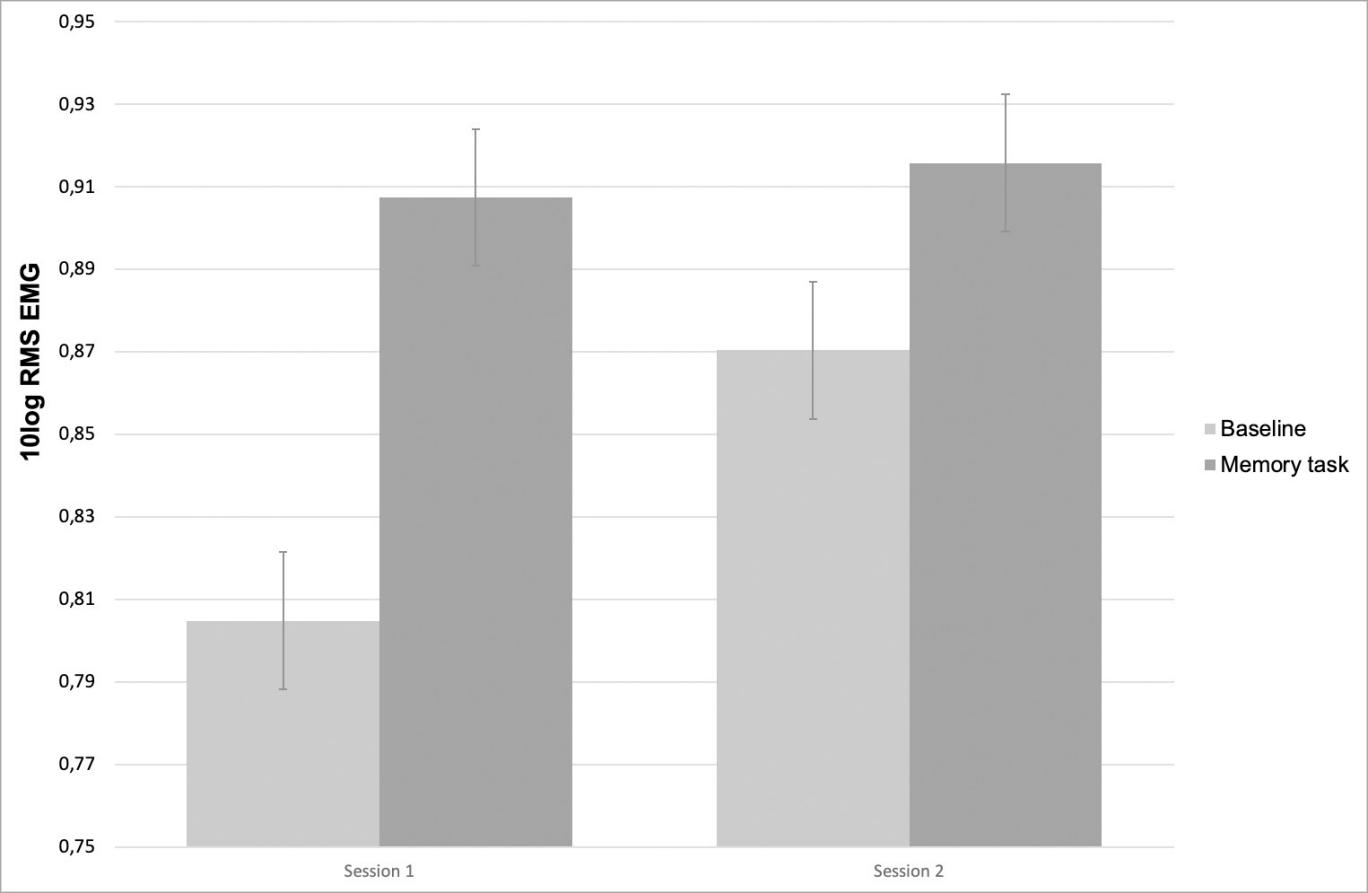
Predicted mean EMG (10log RMS) of the right trapezius muscle. Mean EMG was higher during the memory task at both sessions. Task-reactivity was greater at session 1 compared to session 2 (condition*session interaction significant p < .001).

With respect to the second validation hypothesis, we investigated whether there was a condition*session interaction, indicating lower EMG reactivity at session 2 compared to session 1 [12]. A significant interaction was found for both LTM (t = -7.197, p < .001) and RTM (t = -5.763, p < .001). Post-hoc, it was investigated whether baseline level EMG differed between the two sessions, as suggested in Fig 1. However, this was not the case, as indicated by a non-significant association with session for both LTM (p = .548) and RTM (p = .072). Results of significant associations related to EMG are displayed in Table 2.

**Table 2:** Significant associations with EMG of the trapezius muscle.

		ß	SE	df	t	p-value
**EMG LTM**	Task	.066	.017	108.058	3.861	< .001
	Age	.079	.037	100.129	2.145	.034
	Higher education	.131	.050	98.839	2.608	.011
	Condition*session	-.072	.010	2428.676	-7.197	< .001
**EMG RTM**	Task	.076	.016	106.837	4.788	< .001
	Age	-.002	.001	98.019	-1.664	.099
	Higher education	.109	.053	97.452	2.062	.042
	Condition*session	-.057	.010	1874.213	-5.763	< .001

LTM, left trapezius muscle; RTM, right trapezius muscle; Task coded as 0 = baseline, 1 = memory task.

As described in the method section, EMG data were 10log-transformed. In order to calculate the EMG condition effect in its original (untransformed) scale, a back-transformation was performed. In its original scale, EMG task reactivity was 2.1 times larger at T_0_ than at T_1_ for RTM and 3.59 times for LTM.

Finally, all models showed a significant random intercept, random condition and random session effect (all p < .001), indicating that participants differed regarding general (task irrelevant) EMG variability as well as task reactivity (condition effect) and additionally displayed between session variability.

### The interaction between ACE and EMG reactivity over time

It was investigated whether the condition*session effect was moderated by ACE score. For each ACE score (early childhood and adolescence) we ran a third-order interaction model with a condition*session*ACE score interaction variable. All models were adjusted for the set of seven possible confounders as described in the method section. SPSS syntax for early childhood ACE is depicted in S1 Fig.

The third-order interaction for LTM was significant for both the model with the early childhood ACE score (ß = -.018, t = -3.269, p = .001) and the adolescent ACE score (ß = -.017, t = -2.965, p = .003). For RTM no significant (all p-values > .711) third-order interactions were found. Post-hoc, we investigated whether excluding left-handed participants (N = 6) meaningfully changed the results of the analyses. This was not the case.

**Fig 2:**
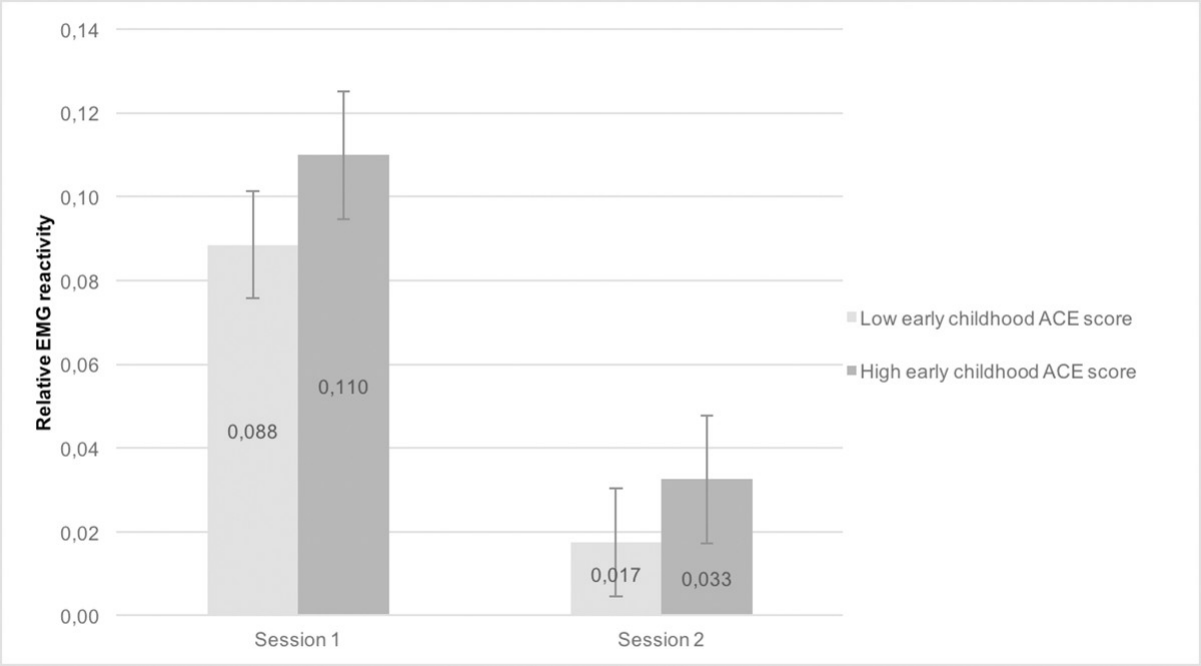
Task reactivity at session 1 and 2, comparing high and low early childhood ACE score categories. Task reactivity was stronger in high ACE group compared to the low ACE group (session*condition effect significant < .001).

**Fig 3:**
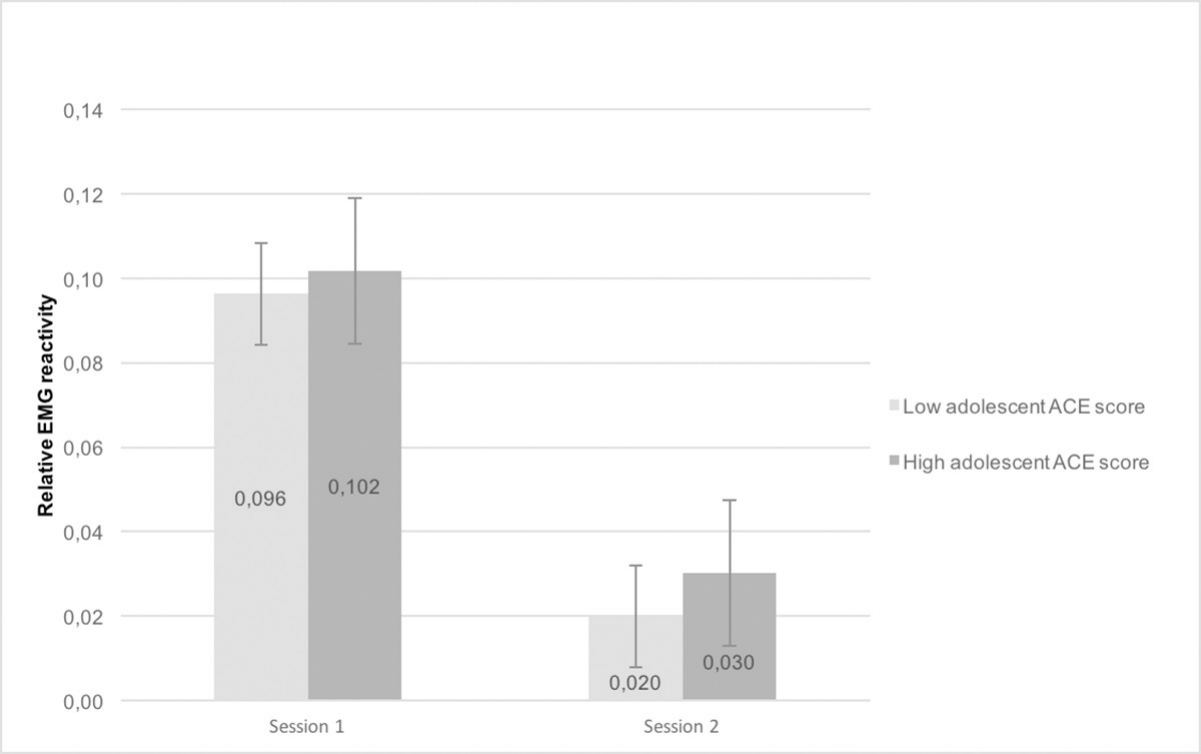
Task reactivity at session 1 and 2, comparing high and low adolescent ACE score categories. Task reactivity was stronger in high ACE group compared to the low ACE group (session*condition effect significant < .001).

In order to further explore the meaning of the third-order interaction, each ACE score was dichotomized at the median split (0 = relatively low ACE score, 1 = relatively high ACE score). Subsequently, the second order session*condition model was analyzed, stratified by the binary (early childhood and adolescent) ACE score variables. Figs 2 and 3 show the expected effect in that task reactivity was stronger in the high ACE group compared to low ACE group. For both groups, task reactivity was attenuated during the second session. In all stratified models, the session*condition effect was significant (all p’s < .001) for LTM. Results for RTM were similar (all p’s < .008).

## Discussion

This study investigated the influence of ACE on EMG activity over time in a newly developed stress paradigm. The first objective of the study was to validate the experimental procedure. Compared to baseline, EMG reactivity was significantly higher during the memory task for both the left and the right trapezius muscle. As the baseline condition for recording EMG was sitting still and silent with eyes open and the experimental condition included the memory task, it could be argued that doing any task (i.e., the memory task) other than sitting still (i.e., the baseline condition) may increase EMG activity. However, combining the observed psychophysiological effect with the fact that all participants (subjectively) reported the task as stressful suggests that the memory task can be considered as a stress-inducing experiment. Nevertheless, follow-up should be conducted including a non-stressful control condition in order to definitively conclude that the observed EMG reactivity specifically indexes stress-reactivity.

Consistent with our a priori hypothesis, the condition*session interaction was furthermore found to be significant. Reactivity to the stressor (the memory task) was smaller in session 2 compared to session 1. The logical explanation for this finding is that participants were familiar with the task at session 2 and thus perceived less unpredictability and uncertainty than the first time. This is in accordance with early studies on human stress, demonstrating that controllability and predictability over an aversive event is less arousing compared to absence of these dimensions [31].

The expected effect of reduced EMG activity with increasing age could not be observed, although the association (p = .099) is suggestive of a trend in the hypothesized direction. Post-hoc, it was found that this may be related to the overrepresentation of higher educated people in the study population, possibly confounding the main effect of age.

Further, it is generally known that people differ in their EMG baseline level, as well as in their reactivity to stressors and in their stress reactivity over time [19]. These notions were confirmed in this study given highly significant random intercept, condition and session effects across all analyses. The results indicate that participants differ regarding general (task irrelevant) EMG variability as well as task reactivity (condition effect) and between session variability. In multilevel analysis, main effects are corrected for these so called ‘random’ effects. The findings further support the notion that the memory task used in this experiment can be considered as a valid stress-inducing experimental paradigm.

The second objective was to investigate the generalizability of the finding reported by Luijcks and coworkers [21] that ACE influences EMG stress reactivity in a stress experiment, in this case a memory task. This was demonstrated for the left trapezius muscle for early childhood ACE (0–11 years) as well as ACE during adolescence (12–17 years). The fact that we not only found an effect for early childhood ACE but also for adolescent ACE, is in line with several other findings in the literature, confirming that ACE can have long lasting effects on the individual [1–3].

In accordance with previous research demonstrating a dose-response relationship between ACEs and the risk of subsequent health problems, it was found that participants with higher ACE scores showed increased EMG reactivity relative to those with lower ACE scores [9, 11]. The impact of the stressor thus was greater for people with higher ACE scores. The negative condition*session effect indicates that individuals experienced less EMG reactivity at the second session. The third order interaction tested to what degree this finding was moderated by ACE score, which indeed was the case. Although EMG reactivity during the second session was lower compared to the first, for people with higher ACE scores (both early and adolescent) the impact of the condition*session effect was still greater compared to participants with lower ACE scores.

The third-order interaction may not appear in accordance with the finding by Luijcks and colleagues [21] who did not report such an interaction. However, the reason they did not find a significant third-order interaction is probably related to the fact that in their stress experiment, participants received an unpredictable and uncontrollable electrical painful stimulus at both sessions. Hence, although participants knew what the stress experiment was about, they had no information about the timing nor the intensity of the electroshock that was administered at both sessions. Thus, the unpredictability of the experiment was similar 6 months later. In contrast, the procedure of the memory task was identical during the second session, thus reducing the unpredictability of the experiment.

The results of the present study may be generalized to real life situations as follows: confronted with an unpredictable or uncertain event, individuals who have experienced early adversity may react with stronger EMG reactivity compared to those who did not. However, in situations where there is no or little unpredictability, there may be no difference in stress reactivity between individuals who did or did not experience ACE.

The question may rise to what degree the ACE questionnaire indexes events that are ‘adverse’ or ‘traumatic’. Although much research has been done in this area, definitions of ‘trauma’ and ‘adversity’ used in the literature are not consistent [32]. A widely used definition of trauma, offered by the American Psychiatric Association, is: exposure to actual or threatened death, serious injury, or sexual violence, either by directly experiencing or witnessing such events or by learning of such events occurring to a close relative or friend [33]. However, other approaches widen the concept of trauma, for instance the definition of Shapiro describing trauma as any event that has had a lasting negative effect upon self and psyche [34]. This definition offers a more dimensional view of adversity, where trauma is positioned at the extreme end [35]. McLaughlin proposed that childhood adversity should be defined as “experiences that are likely to require significant adaptation by an average child and that represent a deviation from the expectable environment” [32]. Therefore, whereas traumatic events taking place in childhood may represent childhood adversity, not all types of childhood adversity may be considered traumatic. ‘Non-traumatic’ ACEs are for example poverty and the absence of a stable, supportive caregiver.

Taken together, the results of the current investigation can be taken to reflect the impact of ACE reflecting childhood adversity and not severe traumatic events at the extreme end of the distribution.

### Limitations of the study

As noted earlier, follow-up research including a non-stressful control condition is required in order to definitively conclude that the observed EMG reactivity indexes stress-reactivity. Furthermore, a future improvement of the paradigm would be to also assess the subjective level of experienced stress during the experiment, for instance using a visual analogue scale (VAS). In the present study, this did not happen.

EMG was measured by two electrodes: one on the right trapezius muscle and one on the left. In future research, it may be productive to place more electrodes and/or attach electrodes to different body parts.

ACE was measured with a self-report questionnaire, which is known to be prone to several forms of bias such as recall bias and response bias. In the case of bias, effects may be underestimated or overestimated. However, whether in this case a structured interview would lead to more accurate results than a self-report questionnaire is not known, since it can entail the same types of bias [36].

In future research, it may be productive to explore the timing of ACE in more detail; the two exposure periods used in this study (0–11 years and 12–17 years) are quite broad and arguably arbitrary. Assessing the experienced impact of ACE would furthermore be of value; the questionnaire used for the current experiment provided no information on this.

It could be argued that the ACE questionnaire is very non-specific regarding the type of event and thus not a good tool to assess the impact of adverse events. We are aware of the fact that many factors contribute to the impact on an individual. In the end, what is considered ‘adverse’ or ‘traumatic’ is highly subjective and therefore nearly impossible to capture with a standardized measure. However, as already pointed out in the introduction, ACEs often co-occur and arguably are best viewed as a complex set of highly interrelated experiences rather than stand-alone events [13]. In our study population, this also seems to be the case, given the fact that approximately 40% experienced two or more ACEs (Table 1). Hence, the scale is thought to be adequate for use in the present study.

The assessment of recent life stressors was very limited and provided no information about the number of events or the experienced impact of the event. In future research, it is recommended to also collect this information.

All validation analyses yielded comparable results for both left and right trapezius muscle reactivity. However, the third-order interaction was significant only for the left trapezius muscle. The difference between the highly significant LTM non-significant RTM effects is of interest. Although we do not have a post-hoc explanation for this finding, there may be an underlying mechanism which requires further elucidation. Therefore, replication of the findings of the present study is required.

### Directions for future research and implications

The memory task is straightforward and easy to perform, independent of personal factors like age, educational level and illness status. The presented paradigm may thus be used in future studies investigating the influence of ACE on stress and (mental) health.

This study was conducted in a general population sample. Examining specific clinical populations (e.g. people diagnosed with PTSD, anxiety disorder or mood disorder) may provide more insight into the relationship between ACE, EMG stress reactivity and (mental) health problems. Furthermore, the memory task may also be used in a clinical setting, in order to assess psychophysiological stress reactivity in relation to treatment and prognosis.

Finally, in clinical practice, one of the issues that remains to be resolved is why certain individuals are resilient after exposure to adversity and others are not. The outcome of this study, as well as the study by Luijcks and colleagues [21], suggests that differential resilience may be investigated using EMG reactivity as a stress marker.

## Supporting information

S1 Data EMG data for multilevel data analyses. (SAV)

S1 Fig SPSS syntax third-order interaction model for early childhood ACE score. (TIF)
