# Supplementary figures and images for: The impact of adverse childhood experiences on EMG reactivity: A proof of concept study
Source: PLoS One. 2019 May 9;14(5):e0216657. doi: 10.1371/journal.pone.0216657 (PMC6508727; doi:10.1371/journal.pone.0216657)

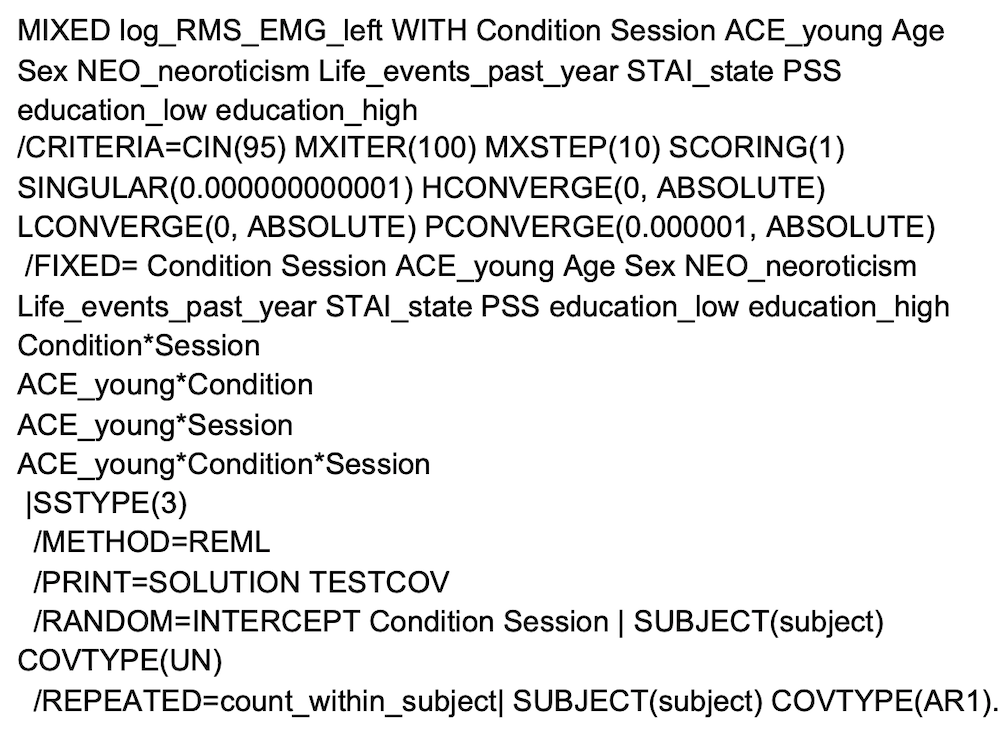

Supplement: S1 Fig — (TIF) [file pone.0216657.s002.tif]
